# Analysis of cognitive framework and biomedical translation of tissue engineering in otolaryngology

**DOI:** 10.1038/s41598-023-40302-6

**Published:** 2023-08-18

**Authors:** Javier Padilla-Cabello, Jose A. Moral-Munoz, Antonio Santisteban-Espejo, Antonio Velez-Estevez, Manuel J. Cobo, Miguel A. Martin-Piedra

**Affiliations:** 1 Program of Biomedicine, University of Granada, Granada, Spain; 2 Department of Otorhinolaryngology, Hospital Universitario Torrecardenas, Almeria, Spain; 3 Department of Nursing and Physiotherapy, University of Cadiz, Cadiz, Spain; 4 Biomedical Research and Innovation Institute of Cadiz (INiBICA), Cádiz, Spain; 5 Department of Pathology, Puerta del Mar University Hospital, Cádiz, Spain; 6 Department of Medicine, University of Cadiz, Cadiz, Spain; 7 Department of Computer Science and Engineering, University of Cadiz, Cádiz, Spain; 8 Department of Computer Science and Artificial Intelligence, Andalusian Research Institute in Data Science and Computational Intelligence (DaSCI), University of Granada, Granada, Spain; 9 Tissue Engineering Group, Department of Histology, University of Granada, Granada, Spain

**Keywords:** Regenerative medicine, Tissue engineering, Translational research

## Abstract

Tissue engineering is a relatively recent research area aimed at developing artificial tissues that can restore, maintain, or even improve the anatomical and/or functional integrity of injured tissues. Otolaryngology, as a leading surgical specialty in head and neck surgery, is a candidate for the use of these advanced therapies and medicinal products developed. Nevertheless, a knowledge-based analysis of both areas together is still needed. The dataset was retrieved from the Web of Science database from 1900 to 2020. SciMAT software was used to perform the science mapping analysis and the data for the biomedical translation identification was obtained from the iCite platform. Regarding the analysis of the cognitive structure, we find consolidated research lines, such as the generation of cartilage for use as a graft in reconstructive surgery, reconstruction of microtia, or the closure of perforations of the tympanic membrane. This last research area occupies the most relevant clinical translation with the rest of the areas presenting a lower translational level. In conclusion, Tissue engineering is still in an early translational stage in otolaryngology, otology being the field where most advances have been achieved. Therefore, although otolaryngologists should play an active role in translational research in tissue engineering, greater multidisciplinary efforts are required to promote and encourage the translation of potential clinical applications of tissue engineering for routine clinical use.

## Introduction

Tissue engineering (TE) is a discipline whose main objective is the development of artificial tissues and organs for the replacement and regeneration of damaged or absent tissues in the patient^1^. Therefore, it allows the procurement of living tissues, implantable and integrable in the patient, with no donor and without the comorbidity derived from obtaining tissue from other parts of the body of the same patient. TE has undergone a fast evolution, especially in recent years^2,3^, leading to a process of consolidation of research and the need for synthesis^4^. Thus, once consolidated, it is important to objectively evaluate the main goal of TE: the development and translation of new therapeutic applications that complement and improve those currently available, constituting Advanced Therapies Medicinal Products (ATMP)^5^.

The development of ATMP by TE acquires a special relevance in the case of patients with craniofacial pathologies since the morbidity derived from the pathology itself is associated with a probable facial aesthetic component, either because of the disease itself or the approach taken for its treatment^6^. In this sense, otolaryngology, as a medical-surgical specialty of reference in head and neck surgery, can assume the different lines of application of TE techniques in this area^7^. Therefore, it interests evaluate the degree of translation of TE to a clinical specialty such as otolaryngology.

Previous studies have attempted to characterize the advances of TE in otolaryngology^8,9^, analyzing the current concepts of TE and its potential applications in the different areas of this medical specialty. These papers report descriptive analyses of the current situation of TE in otolaryngology. However, there is a lack of systematic analyses aimed at evaluating the cognitive framework of TE and otolaryngology through the use of knowledge-based tools, such as science mapping analysis (SMA). Using SMA allows the characterization of the cognitive structure of this research topic^10,11^. The application of SMA software makes a series of relevant topics emerge and classify them based on their external and internal coherence of the different themes constituting the thematic network^12,13^. The analysis of the thematic networks makes it possible to understand the origin of these themes and the terms that make them up, thus generating plausibility and a logical interpretation of the results obtained.

Furthermore, there is no previous analysis about of the translation of this research area. In that sense, an adequate assessment of the degree of clinical translation of a discipline, such as TE, is difficult to achieve. In this context, a recent systematic review gathers all the methods for assessing the degree of translation of a discipline available in the literature^14^, describing their advantages and limitations. One of the first methods described for this purpose is the Triangle of Biomedicine^15^, the base for the current method used to evaluate translation in National Institute of Health (NIH) databases. These methodologies could be potentially useful to systematically evaluate the degree of translational TE in medical specialties, such as otolaryngology. This translational evaluation allows to assess, among other aspects, whether the discipline is being developed, which lines of research or which areas are more advanced, where it is worth investing, or whether TE is being applied in any field of daily clinical practice. The assessment of translational research productivity through bibliometric analysis is becoming increasingly important among funding agencies^16^. That is why establishing a system for evaluating the translation of an emerging discipline into a clinical specialty would be a standard for evaluating the benefit of these investments^17,18^.

In view of this background, the main aim of this work is to evaluate the cognitive framework and biomedical translation of the TE and otolaryngology research area, providing a wider perspective on the development of new advanced therapies in this clinical specialty. For this purpose, different knowledge-based approaches, such as the science mapping analysis and the identification of the biomedical translation through the biomedical triangle, were used.

## Material and methods

### Sample

The set of documents of TE and otolaryngology applications was retrieved from the Web of Science (WoS) core collection database of Clarivate Analytics (London, United Kingdom). A topic search was used to retrieve all the papers on TE belonging to otolaryngology, using the query TS = ("tissue engineer*" OR "tissue-engineer*") AND WC = Otorhinolaryngology for the period between 1900 and 2020. This query is based on previous analyses focused on TE^2,3^.

### Science mapping analysis

Scientific mapping allows us to visually assess the relationships established between documents thanks to the spatial representation established between the different nodes^10^. SMA is considered an important area in bibliometrics^11^. To perform this type of analysis, we used SciMAT (Science Mapping Analysis Tool), a freely available bibliometric software that allows the construction of scientific maps based on the co-occurrence of keywords^19^.

Keywords from documents were loaded to SciMAT, creating a single time period (1994–2020) and submitted to three different stages, as previously described^2^. Briefly, a normalization of authors and words by singulars, plurals and Levenshtein distances was applied. Keywords are clustered in conceptual nodes, named themes. Then, networks based on the co-occurrence of the unit of analysis (keywords clustered on themes) were constructed. Once networks were developed, each of the obtained themes was plotted on a low dimensional space layout based on Callon’s centrality and density^12,13^. These indicators allow to classify each theme into four quadrants:

1. *Motor themes* present a strong density and high centrality, and show the most intensely developed topics in the research area of interest (upper right quadrant).
2. *Basic and transversal themes* represent themes shared by several disciplines, so their fundamentals are well defined (lower right quadrant).
3. *Emerging or declining topics* they have a weak density and low centrality, thus signifying marginal areas of knowledge (lower left quadrant). The differentiation between emerging and declining themes requires a prospective assessment of their centrality.
4. *Highly developed or isolated themes* show a high density showing significant internal development. However, they are less connected with other themes in the research field because of their low centrality values (upper left quadrant)

Finally, the number of documents and citations received were used as bibliometric indicators to analyze the thematic areas.

### Biomedical translation analysis

In order to get the information about the biomedical translation, iCite (https://icite.od.nih.gov) translation module was used on the set of documents previously identified. This module assigns each document a score in three different categories (Human, Animal and Molecular/Cellular Biology) based on the number of Medical Subject Heading (MeSH) terms. These scores represent the focus of the article content on these dimensions. The papers are represented in the "translational space" of the Triangle of Biomedicine^15^. Articles with high scores in "Human" will appear closer to the Human vertex, while those with high scores in "Animal" and "Molecular/Cellular Biology" will appear closer to those respective vertices.

This module also uses this information to track and predict citations for clinical articles by calculating the Approximate Potential to Translate (APT). This is an estimate obtained by a machine-learning algorithm, with a value of 0, 25, 50, 75, 95 or 100%, which shows the probability that a document will be cited by a clinical article^20^. Furthermore, using the themes detected in the SMA, a ternary plot was obtained, following the Triangle of Biomedicine principles. The values for each category, Human, Animal and Molecular/Cellular, were calculated for each of the detected themes. Then, using Python programming, these values were represented by spheres in a ternary plot. The volume of the spheres is based on the number of documents that compound the theme and this value is also shown. Finally, two different areas were established to represent the clinical and basic research, the first one composed of those topics closer to the Human vertex (colored blue), and the second one closer to the Animal and Molecular/Cellular Biology categories (colored orange).

## Results

### Sample

A total of 343 documents were retrieved from WoS after performing the search strategy described above, corresponding to 26 years (1994–2020) of global production that form the corpus of literature at TE in otolaryngology.

### Science mapping analysis

**Figure 1:**
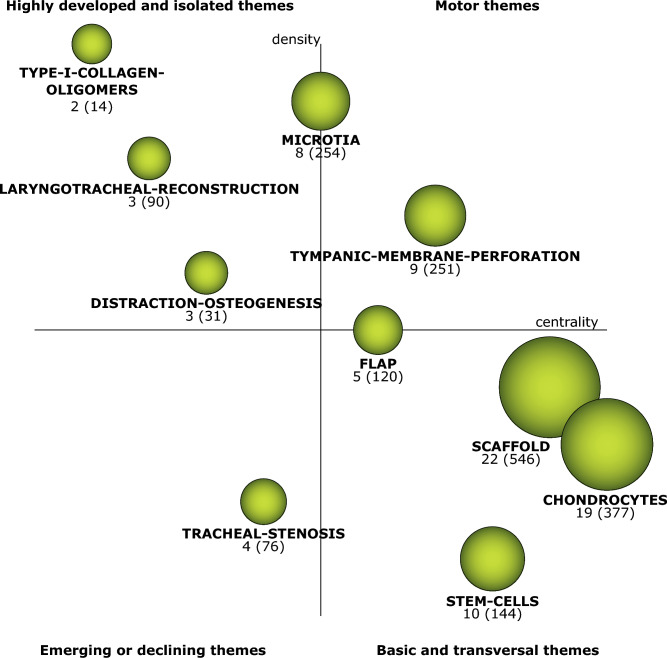
Strategic diagram for the conceptual framework of tissue engineering in otolaryngology.

**Figure 2:**
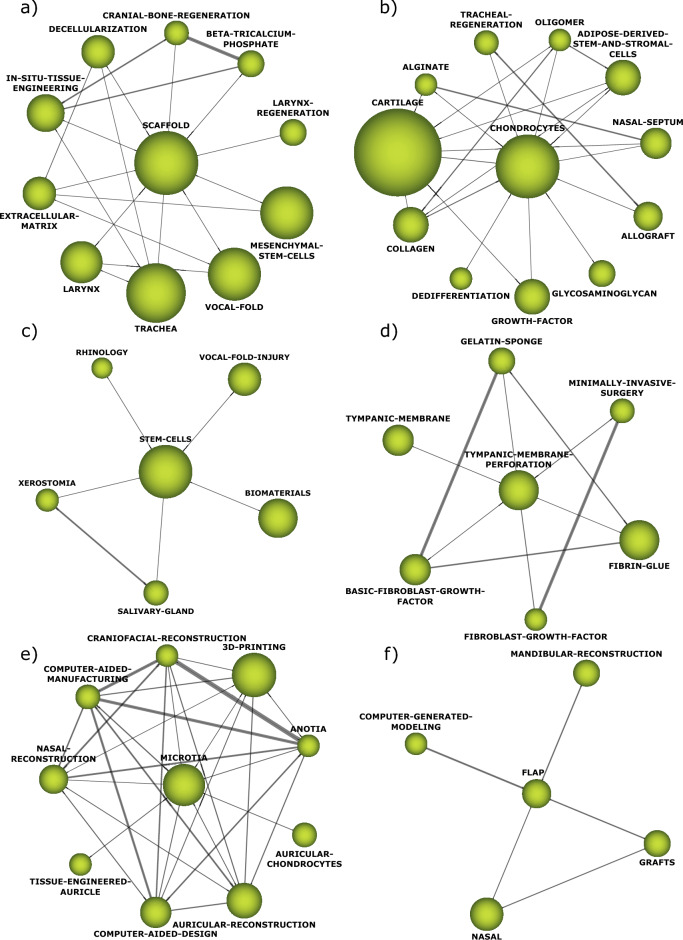
Thematic networks of the basic or transversal themes (**a**–**c**): (**a**) Scaffold thematic network; (**b**) Chondrocytes thematic network; (**c**) Stem-cells thematic network. Thematic networks of the motor themes (**d**–**f**): (**d**) Thematic network of tympanic-membrane-perforation; (**e**) Thematic network of microtia; (**f**) Thematic network of flap.

The analysis performed by SciMAT shows the themes of interest in TE in the field of otolaryngology research (Fig. 1). First, those themes considered basic or transversal were described, followed by the motor themes. Third, highly developed and isolated themes were analyzed, and finally, those that were emerging or declining. In each of these four cases, the concepts that make up each cluster were presented. Within the basic or transversal themes, we found those themes that brought together the largest number of documents and citations received, with the term *scaffold* occupying first place, followed by *chondrocytes* and *stem-cells*. Furthermore, three themes were identified as motor themes, corresponding to *tympanic-membrane-perforation*, *microtia,* and *flap*. Figure 2 shows the thematic networks of the basic and transversal and motor themes.

**Figure 3:**
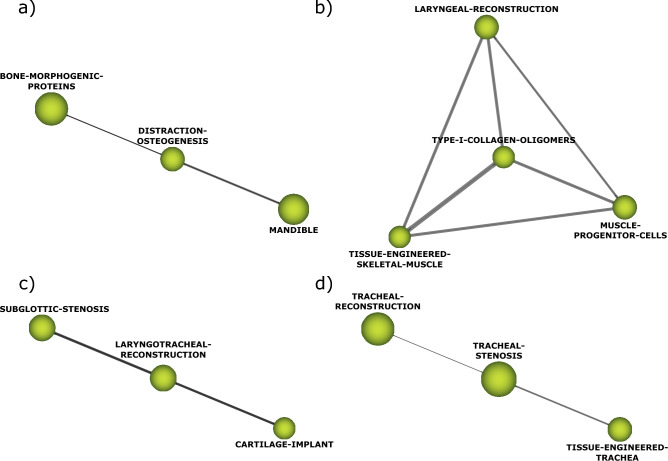
Thematic network of highly developed and isolated themes (**a**–**c**): (**a**) Thematic network of distraction-osteogenesis; (**b**) Thematic network of type-I-collagen-oligomers; (**c**) Thematic network of laryngotracheal-reconstruction. (**d**) Thematic network of the emerging or declining theme tracheal-stenosis.

Both highly developed or isolated themes and emerging or declining themes are characterized by low centrality (below 0.5). The themes *distraction-osteogenesis*, *type-I-collagen-oligomers* and *laryngotracheal-reconstruction* were identified as the main highly developed or isolated themes, while *tracheal-stenosis* was the only theme considered emerging or declining. Figure 3 shows the thematic networks of the highly developed or isolated themes and emerging or declining themes.

### Biomedical translation

iCite tool uses information obtained from PubMed, so the PMIDs of 331 documents were obtained (there is a loss of 12 records, which represents 3.49% of the total). This application categorizes only nine documents as clinical articles (2.72%) corresponding to clinical trials or clinical guidelines.

**Figure 4:**
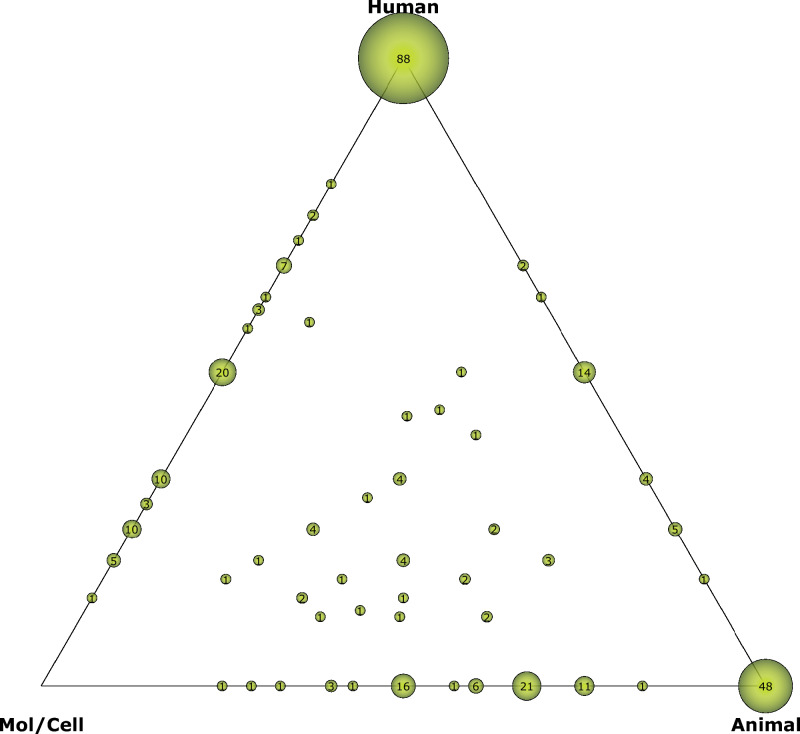
Bubble plot showing the distribution of the set of documents in the Triangle of Biomedicine.

The different scores generated in the iCite translation module were obtained from the total number of documents and clinical articles (Table 1). From the total number of documents, it can be observed that the average “Human” score is 0.41, “Animal” score is 0.35 and “Molecular/cellular” score is 0.23. The distribution of the articles according to these scores is shown in Fig. 4. The average APT of this set of analyses is 25.4%. Only 79 papers (23.86%) have been cited, at the time, by clinical articles. The two papers that received the most citations from clinical articles were a non-clinical article, with 13 citations^21^, and one clinical article that received a total of 12 citations^22^, both aimed to tympanic perforation closure. Table 2 shows the five most cited by clinical articles, if it is a clinical article or not, its main theme, and the number of citations it has received.

**Table 1:** Average scores and APT obtained from the translational module for the total publications and clinical articles.

	Avg. Human	Avg. Animal	Avg. cell/molecular	Avg. APT	Cited by a clinical article
Total publications	0.41	0.35	0.23	25.4%	79
Clinical articles	0.96	0	0.04	52.2%	5

**Table 2:** Top-cited documents referred to tissue engineering in otolaryngology by citation count.

Reference	Article type	Theme	Citations count by clinical articles
Hakuba et al*.*^21^	Non-clinical	Tympanic perforation closure	13
Kanemaru et al.^22^	Clinical	Tympanic perforation closure	12
Kelley et al*.*^23^	Non-clinical	Microtia	4
Silveira et al*.*^24^	Clinical	Tympanic perforation closure	4
Kanemaru et al*.*^25^	Non-clinical	Vocal fold regeneration	3

**Figure 5:**
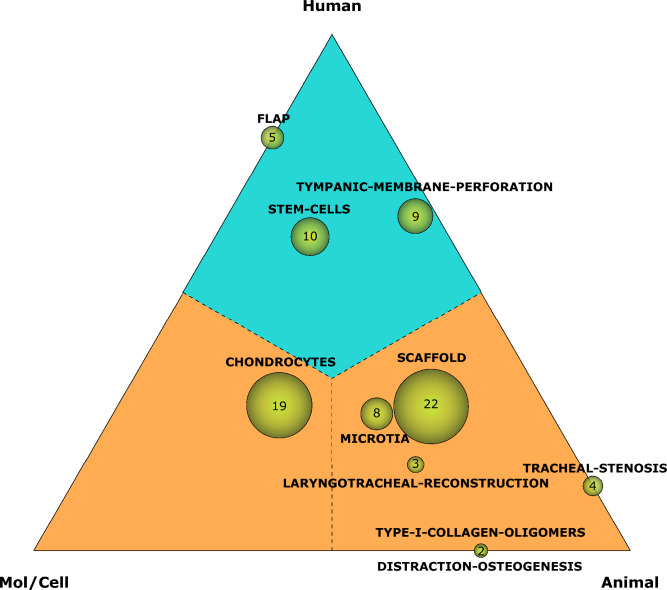
Triangle of Biomedicine with the distribution of the different themes identified in the SMA. This triangle is segmented into two areas according to the nearest vertex. The basic research area (orange) is the area whose nearest vertices are Mol/Cell or Animal. The clinical research area (blue) is the area of the triangle whose nearest vertex is Human.

When performing the analysis of the translation according to the different themes identified by SciMAT and distributing them into the Triangle of Biomedicine, it is possible to observe those that have a position closer to the human and those topics that are still in an intermediate phase or closer to the animal vertex. As presented in Fig. 5, three groups of themes can be identified according to their location within the triangle. In the first place, those themes located at a central position are located intermediately between the three vertices. This would be the case of the basic or transversal themes *chondrocytes* and *scaffold*, together with the motor theme *microtia* and the highly developed theme *laryngotracheal-reconstruction*. The set formed by the themes *tracheal-stenosis, type-I-collagen-oligomers* and *distraction-osteogenesis*, the latter two highly developed and isolated themes, are in a more displaced position to the animal vertex. These two groups do not present Human as the nearest vertex, thus they were considered as basic research (orange). Finally, the themes *flap*, *stem-cells* and the main motor theme, *tympanic-membrane-perforation*, are found in the upper segment of the triangle, presenting the human vertex as the nearest vertex and being considered as clinical research (blue).

## Discussion

TE is an area of research aimed at the development of artificial tissues and organs that can restore, maintain or even improve the anatomical and/or functional integrity of injured tissues^1^. Although it is a relatively young discipline, recent studies have shown a trend towards its consolidation through the bibliometric analysis of its scientific production^2–4^. Therefore, the time is ripe to evaluate whether the advances achieved in this area of research are applied in clinical practice, which is known as biomedical translation. This work tries to quantitatively assess the cognitive structure of TE in otolaryngology, as well as estimate its degree of translation for the first time in the biomedical literature.

Different knowledge-based methods to perform this evaluation have been described in literature^14^. Using SMA allows the characterization of the cognitive structure of TE in otolaryngology. The main tool used in this work was the analysis of science maps based on the co-occurrence of keywords, which allowed us to discover a series of complex relationships between the different concepts (themes) in the area of study. The application of software such as SciMAT makes a series of relevant themes emerge and classify them on the basis of their centrality and density, which are bibliometrics indicators measuring the external (inter-node) and internal (intra-node) coherence of the different themes (nodes) constituting the thematic network that lead to a more logical and plausible interpretation of results. Particularly noteworthy is the presence of three transversal themes in this area, such as *scaffold*, *chondrocytes* and *stem-cells*.

The search for a scaffold with adequate biomechanical characteristics is an area of interest in different fields, for example, in the reconstruction of the larynx, trachea or for the repair of cranial bone defects^26,27^. All these concepts are linked to the central term scaffold, together constituting the *scaffold* theme. In the case of cranial bone defect repair, there is a significant link between this theme, the use of tricalcium beta-phosphate and the application of in situ TE. This connection derives from the application of matrices composed of tricalcium beta-phosphate for the induction of in situ formation of new bone for the closure and repair of cranioencephalic bone defects^28,29^. This theme occupies a central position in the basic research area of the Triangle of Biomedicine, which shows its profile as a basic and transversal theme shared by several disciplines.

The second of the themes highlighted in the cognitive structure of the analyzed corpus was *chondrocytes*. The procurement of chondrocytes and the subsequent generation of cartilage tissue through TE is a basic research topic, being the only theme found in the area of basic cellular/molecular research in the Triangle of Biomedicine. TE of cartilage is a fundamental aspect in the development of grafts for the reconstruction of structures made up of a cartilaginous skeleton such as the trachea or nasal septum, both terms present in the thematic network of *chondrocytes*^30,31^. Likewise, this network includes some of the most important points from the perspective of TE in the generation of cartilage tissue. Adipose tissue stem cells also appear in this network since they are the most common cells used to differentiate into chondrocytes^32,33^. We also find the two main biomaterials in the development of artificial cartilage, which are collagen and, above all, alginate^34,35^ as its properties closely resemble those of the native cartilage matrix^36,37^.

Finally, the set defining the *stem-cell* thematic network also proved to be a cross-cutting theme in this study. A link was observed with the themes rhinology and vocal fold injury, given the potential use of stem cells in the regeneration of nasal cavity mucosa^38,39^ or altered vocal folds^40^. There is also a relationship with the salivary glands and xerostomia, and a strong link between these two subjects, since one of the main applications being investigated is their use for xerostomia, especially in patients who have received radiotherapy in the head and neck area^41,42^. Interestingly, its position within the human area in the triangle of medicine suggests that possible clinical applications of stem cell use have been tested.

The theme related to *tympanic-membrane-perforation* emerged as the main motor theme. Tympanic perforation is a prevalent pathology, mainly caused by trauma or repeated otitis media^43^, which is associated with constant hearing loss or otorrhea^44,45^. This comorbidity, although mild, significantly affects the quality of life of patients. Surgical closure of these by tympanoplasty surgery has a variable and inconsistent success rate^46^. In the network of this theme are elements and scaffolds, such as fibrin glue or gelatin sponge, and fibroblast-derived growth factors obtained. Using these compounds obtained by TE techniques is postulated as alternatives to conventional surgery for minimally invasive closure of tympanic perforations^47,48^. Another finding that confirms the important role of tympanic membrane perforation research in TE in the field of otolaryngology is the fact that, of the nine papers that were identified as clinical papers according to iCite, five dealt with this clinical problem^22,24,47–49^. This is another theme that falls within the human area in the Triangle of Biomedicine. As we have seen, it is one of the main topics of TE research within otolaryngology, constituting the research objective of the main clinical articles. This not only reflects the great interest in this field of study, but also the driving force to promote clinical translation.

One of the best established themes was *microtia* as it showed high Callon’s density. *Microtia* theme lies on the borderline between highly developed and motor themes, with a high number of nodes in its thematic network. This is because the creation of artificially created pinnae for the reconstruction of microtia or anotia is one of the most popular and well-known research topics in otolaryngology^50,51^, with the milestone of the regeneration of a tissue-engineered human pinna in the back of a rat in 1997^52^. This theme can be associated with *chondrocytes*, but in this case it is specifically investigated with auricular chondrocytes for the generation of cartilage similar to that present in an auricle^53,54^. It can also be extracted from interpreting the thematic network that computer design and 3D printing of these TE products play an important role in this research topic^55^. However, despite all these advances, this is a subject that occupies a central position in the triangle of biomedicine, which shows that its translation to humans has not yet been applied. Other clinical themes like *tracheal-stenosis* or *laryngotracheal-reconstruction* are in a more displaced position to the animal vertex, showing that they also are still far from a human application phase. Despite this, their character as emerging and highly developed themes, respectively, may be an indication that laryngotracheal reconstruction techniques for pathology such as tracheal stenosis, using TE techniques, may in the future occupy a place in the area of human clinical research with possible clinical applications.

Despite the results presented in this study could be of interest to the research community in TE and otorhinolaryngology, some limitations need to be addressed. First, we have used the citation count as a proxy of scientific impact. The fact that one paper cites another is an indication that the cited paper has had some influence, or impact, on the paper citing it. However, the authors can cite a paper because of different reasons (to give credit to their mentors, to reinforce an idea or to discuss their findings with the previous literature). As stated by Garfield and Belter^56,57^, which is true is that highly cited papers are highly useful to authors for writing other papers, but what those papers are useful for, however, is not clear. Citation count, as all the bibliometric indicators (i.e. journal impact factor, H-index, etc.) need to be used responsibly, and following the international recommendations^58,59^. Last, documents were obtained from the Core Collection database of WoS, which could include certain limitations, such as language bias or limited inclusion of grey literature (thesis dissertations, patents or conference abstracts), which can be important sources of information. However, WoS includes over 21,000 peer-reviewed, high-quality scholarly journals published worldwide (including Open Access journals) in over 250 disciplines, and is considered a reference by the scientific community as one of the most complete and reliable databases of scientific information^60,61^.

In conclusion, TE is still in an early translational stage in otolaryngology. Regarding the analysis of the cognitive structure, we find consolidated research lines, such as the generation of cartilage for use as a graft in reconstructive surgery, reconstruction of microtia or the closure of perforations of the tympanic membrane. This last research area occupies the most relevant clinical translation with the rest of the areas presenting a lower translational level, placing otology as the field with the most advances in TE in otorhinolaryngology. Therefore, although otolaryngologists and head and neck surgeons should play an active role in translational research in TE, greater multidisciplinary efforts are required to promote and encourage the translation of potential clinical applications of TE for routine clinical use.

## Data Availability

The datasets generated during and/or analysed during the current study are available from the corresponding author on reasonable request.
